# Is hepatic artery coil embolization useful in distal pancreatectomy with en bloc celiac axis resection for locally advanced pancreatic cancer?

**DOI:** 10.1186/s12957-019-1667-8

**Published:** 2019-07-17

**Authors:** Atsuhiko Ueda, Nozomu Sakai, Hideyuki Yoshitomi, Katsunori Furukawa, Tsukasa Takayashiki, Satoshi Kuboki, Shigetsugu Takano, Daisuke Suzuki, Shingo Kagawa, Takashi Mishima, Eri Nakadai, Masaru Miyazaki, Masayuki Ohtsuka

**Affiliations:** 1 Department of General Surgery, Graduate School of Medicine, Chiba University, 1-8-1 Inohana, Chuo-ku, Chiba, 260-8670 Japan; 2 Surgery and Digestive Disease Center, International University of Health and Welfare, Mita Hospital, Tokyo, Japan

## Abstract

**Background:**

The exact contribution of preoperative coil embolization in distal pancreatectomy with en bloc celiac axis resection (DP-CAR) for the prevention of ischemic liver complication is not fully elucidated.

**Methods:**

From January 2004 to July 2015, 31 patients underwent DP-CAR for the pancreatic body–tail cancer. Twenty-three patients received preoperative coil embolization. The characteristics and operative outcomes were analyzed retrospectively.

**Results:**

The median survival time and 1- and 3-year overall survival rates were 23.7 months and 74.2% and 34.4%, respectively. No 30-day mortality occurred in any of the patients. Postoperative liver infarction developed only in 8 patients (25.8%) even though 7 of 8 patients had undergone preoperative coil embolization. Tumor contact with the gastroduodenal artery (GDA)/proper hepatic artery (PHA) on preoperative multi-detector computed tomography (MDCT), tumor size, operative time, portal vein resection, and stenosis of the GDA/PHA after DP-CAR are related to liver infarction. Among them, postoperative stenosis of the GDA/PHA on MDCT, which was observed in all 8 patients with liver infarction, was the most closely related factor to postoperative liver infarction. Tumor contact with the GDA/PHA did not worsen the R0 resection rate or overall survival rate.

**Conclusion:**

Our data indicate that preoperative coil embolization of the common hepatic artery is not useful in DP-CAR as long as GDA is completely preserved during surgery.

## Introduction

Pancreatic ductal adenocarcinoma (PDAC) is one of the leading causes of mortality. Despite recent advances in chemoradiotherapy [1–5], surgical resection is still mandatory to achieve long-term survival. Locally advanced PDAC easily invades major vasculatures, such as the portal vein (PV), superior mesenteric vein (SMV), common hepatic artery (CHA), celiac axis (CeA), and splenic artery (SPA), because of its location. Therefore, decision-making on whether concomitant major vascular resection is possible or not is important to achieve curative resection, which may ultimately lead to the long-term survival of patients with PDAC.

Historically, the presence of vascular invasion had been judged as a contraindication for surgical treatment mainly because of its high morbidity and less favorable effect on long-term survival. However, several high-volume centers have demonstrated that vascular resection can be performed with acceptable morbidity and mortality [6] and is no longer a contraindication for patients with locally advanced PDAC. A previous meta-analysis has demonstrated that arterial resection (AR) is associated with an increased risk of perioperative mortality and poor survival [7]. However, Bachellier et al. reported that pancreatoduodenectomy (PD) with AR can be performed safely and offer a long-term survival compared with PD without AR [8]. Additionally, we have also reported the usefulness of AR in pancreatic resection [9].

Distal pancreatectomy with en bloc celiac axis resection (DP-CAR) is an alternative to improve resectability of locally advanced PDAC, which involves the CeA and CHA [10]. DP-CAR is basically a modified version of the Appleby procedure that was reported in 1953 [11]. Although improved resectability and the favorable long-term outcome can be expected, relatively high rates of morbidity and mortality have been reported [12, 13]. According to a recent international multicenter analysis, the postoperative morbidity and mortality rates of DP-CAR are between 25.0% and 28.0% and 6.0% and 16.0%, respectively [14]. In this procedure, the CeA, CHA, and left gastric artery (LGA), which are major sources of arterial blood flow of the liver and stomach, are resected and are not reconstructed. Therefore, ischemic complications in the liver and stomach are one of its major concerns. To reduce the risk of these ischemic complications, preoperative coil embolization has been reported [15, 16]. The aim of the current study was to assess the role of hepatic artery embolization in preventing the ischemic complication of the liver after DP-CAR.

## Patients and methods

Thirty-one consecutive patients who underwent DP-CAR for the pancreatic body–tail cancer from January 2004 to July 2015 were retrospectively analyzed. This study was approved by the Institutional Ethics Committee of our institution. Informed consent was obtained from all patients after notification of the extent of diseases and the benefits and risks of treatments.

To evaluate the efficacy and feasibility of DP-CAR, the clinicopathological factors, postoperative outcomes, and long-term results were analyzed retrospectively. The surgical indication for DP-CAR was determined according to the following criteria: (1) tumor involvement of the celiac trunk and/or CHA, but not the superior mesenteric artery (SMA), and (2) tumor involvement of the SPA root. Patients with tumor contact with the gastroduodenal artery (GDA) without apparent encasement were included in the criteria. The extension of tumors and vascular involvement were evaluated by contrast-enhanced multi-detector computed tomography (MDCT). Distant metastases were evaluated by EOB-MRI and PET. All findings were evaluated by surgeons and radiologists, and the surgical indication was decided in a multidisciplinary team meeting in our institution.

### Neoadjuvant chemotherapy

Neoadjuvant chemotherapy (NAC) was administered for 24 patients but not for 7 patients who underwent DP-CAR before 2008. Chemotherapy regimen included gemcitabine+S-1 (GS), S-1 alone, and gemcitabine+nab-paclitaxel (GnP). Heavy ion radiotherapy was performed for 2 patients.

### Preoperative coil embolization

**Fig. 1:**
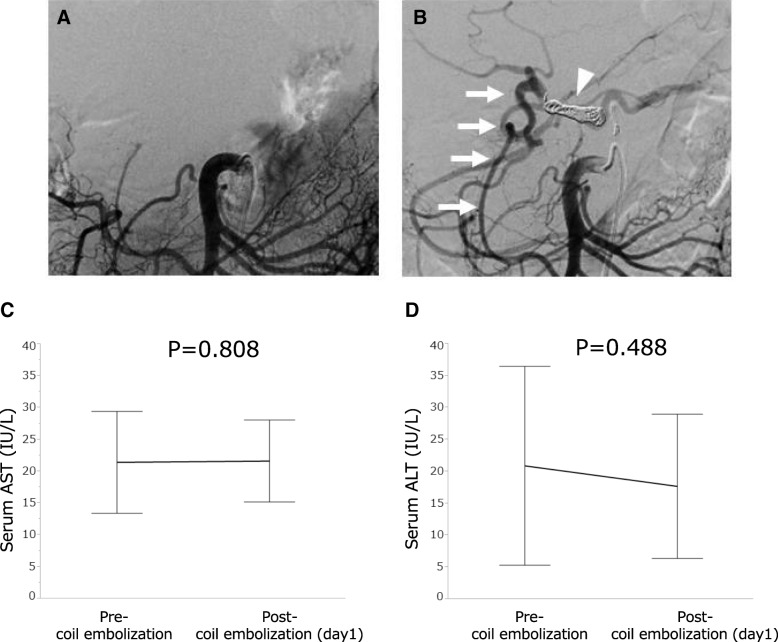
Superior mesenteric arteriogram before (**a**) and after (**b**) CHA coil embolization (white arrowhead). The collateral arterial flow to the liver (white arrow) via the GDA from the SMA was observed in the superior mesenteric arteriogram. Both serum AST (**c**) and ALT (**d**) levels did not increase after CHA coil embolization in 23 patients who underwent this treatment

We introduced preoperative coil embolization in 2008, and therefore, we did not perform preoperative coil embolization for 4 patients who underwent surgery before 2008. Since the introduction, we have performed this technique for 23 patients to enhance collateral arterial blood flow to the liver and stomach through pancreaticoduodenal arcades from the SMA to the GDA, gastroepiploic artery, and right gastric artery around the stomach. Of 23 patients who underwent preoperative coil embolization, NAC was administered for 20 patients. NAC was administered before coil embolization in 12 patients and after coil embolization in 8 patients. Each physician decided the timing of coil embolization. Preoperative coil embolization was not performed for 4 patients who had replaced hepatic artery (i.e., the right hepatic artery branched from the SMA). The procedure was performed according to previous reports with minor modifications [15–17]. Briefly, a 4-Fr catheter was inserted through the right femoral artery. Angiograms of SMA and CeA were performed, and blood flow and extent of tumor invasion in major vessels around the pancreas (i.e., PV, SMV, CeA, CHA, proper hepatic artery (PHA), SMA) were evaluated. Two-Fr microcatheter was then inserted selectively into the CHA through the 4-Fr catheter. The embolization of the CHA was performed using interlocking detachable coils (IDCs, Boston Scientific Japan, Tokyo, Japan; Target, Stryker Japan, Tokyo, Japan). Two to three coils were mostly used to achieve appropriate placement of these coils as an anchor. After the placement of anchoring coils, usual embolization coils were added until the blood flow of CHA was completely occluded. Finally, an angiogram of the SMA was taken to confirm altered arterial blood flow from the SMA to the PHA through the GDA (Fig. 1a, b).

### Definition of liver infarction

We performed contrast-enhanced MDCT at postoperative day 7 to assess postoperative liver infarction and status of the arterial blood flow in the liver. Liver infarction was evaluated according to the following criteria: (1) low-density area on plain CT, (2) no enhancement during any of the phases from arterial to venous phase, and (3) more than 1% of whole liver volume calculated using a 3D-image analysis system (SYNAPSE VINCENT; FUJIFILM Medical Co., Ltd, Tokyo, Japan). We routinely performed contrast-enhanced MDCT to follow up the ischemic lesions every 3–6 months after DP-CAR. Local and distant recurrences were also evaluated in the MDCT. If there were indefinite lesions, MRI or PET was considered depending on the CT findings.

### Statistical analysis

All data were collected retrospectively. A *p* value of < 0.05 was considered significant. Correlations between two categorical variables were assessed using the chi-square test or Fisher’s exact test when appropriate. Estimated survival rates after DP-CAR, including any deaths, were calculated using the Kaplan–Meier method. Statistical analyses were performed with JMP 12 (SAS Institute Japan, Tokyo, Japan).

## Results

### Feasibility of coil embolization

Preoperative coil embolization was performed in 23 patients (74.2%). As shown in Fig. 1a, b, collateral arterial flow to the liver from the SMA to the GDA was clearly confirmed immediately after embolization of the CHA in every case. No significant increase was observed in aspartate aminotransferase (AST) or alanine aminotransferase (ALT) levels 1 day after embolization (Fig. 1c, d). We performed MDCT after the embolization. No embolization-related complications, including transient liver dysfunction, development of liver infarction, and dislodgement of coils, were observed. Arterial blood flow in the liver was also clearly detected on MDCT after the embolization, although precise evaluation of blood flow of the CHA was difficult due to an artifact of coils. Duration form the embolization to DP-CAR was 18 days. All patients who were performed coil embolization underwent DP-CAR.

### Patients’ characteristics and operative outcomes of DP-CAR

Demographic and clinicopathological features of patients are shown in Table 1. Median age of patients was 62.0 years old. Twenty-four patients (77.4%) received NAC. Among 24 patients, 20 patients received GS therapy, 1 patient S-1 therapy, 1 patient GnP therapy, 1 patient GS+ heavy ion radiotherapy, and 1 patient S-1+ heavy ion radiotherapy. Seven patients who did not receive NAC underwent DP-CAR without any other treatments. Twenty-three patients (74.2%) received adjuvant chemotherapy. In terms of liver function, ICG R15 assessed before DP-CAR (i.e., after completion of NAC and coil embolization) was 8.6% (median, 4.9–24.7). Chemotherapy-associated liver injury was not observed in any patients. Median operative time was 334 min, and blood loss was 1270 g. Concomitant PV resection and reconstruction were performed in 16 patients (51.6%). In terms of pathological characteristics, 14 patients (45.2%) had arterial invasion, and 29 patients (93.5%) had a perineural invasion. R0 resection was achieved in 13 patients (41.9%).

**Table 1:** Clinicopathological factors of patients undergoing DP-CAR

Factor	DP-CAR (*n* = 31)
Age (years)	62.0 (39–80)
Gender, male/female	21/10
CEA (ng/mL)	3.5 (0.7–493.0)
CA19-9 (U/mL)	235.5 (0.1–16,860.0)
Neoadjuvant chemotherapy, yes/no	24/7
Preoperative coil embolization, yes/no	23/8
Operative time (min)	334 (175–584)
Blood loss (g)	1270 (305–10,270)
Blood trans fusion, yes/no	17/14
Portal vein resection, yes/no	16/15
Gastrectomy, yes/no	5/26
Bile duct resection, yes/no	1/30
Tumor size (cm)	4.4 (2.0–10.0)
Histologic type, well/moderate/poor/other	10/14/4/3
Lymph node metastasis, yes/no	24/7
Arterial invasion, yes/no	14/17
Portal vein invasion, yes/no	21/10
Perineural invasion, yes/no	29/2
Residual tumor status, R0/R1, R2	13/18
UICC stage (7th edition), IB, IIA/IIB, III, IV	6/25
Adjuvant chemotherapy, yes/no	23/8

All results are shown as median (range) or number

*UICC* Union for International Cancer Control

### Long-term outcome of DP-CAR

**Fig. 2:**
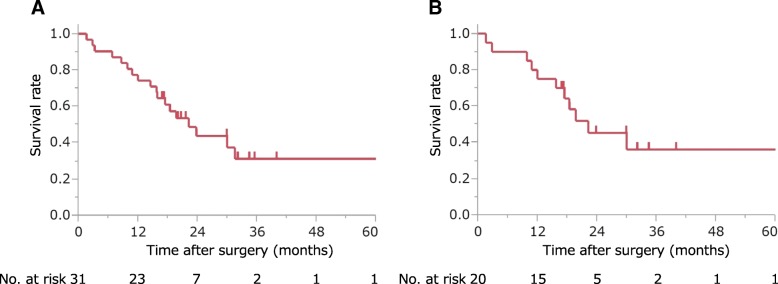
Long-term outcomes of patients who underwent DP-CAR. **a** Overall survival of all patients. **b** Overall survival of patients with PDAC in stages IIB and III classified by UICC staging

The median survival time (MST) and 1- and 3-year overall survival rates after DP-CAR were 23.7 months and 74.2% (95% CI 56.3–86.5%) and 34.4% (95% CI 17.1–57.1%) (Fig. 2a), respectively. In patients with UICC stages IIB and III, the MST and 1- and 3-year overall survival rates were 30.0 months and 75.0% (95% CI 52.2–89.2%) and 41.5% (95% CI 19.6–67.4%) (Fig. 2b), respectively.

### Morbidity and liver infarction after DP-CAR

The morbidity rate of Clavien–Dindo grades (C–D) III to V was 67.7%. No 30-day mortality occurred after DP-CAR (Table 2).

**Table 2:** Surgical outcomes of patients undergoing DP-CAR

Factor	DP-CAR (*n* = 31)	Percent
Morbidity*		
C–D I, II/C–D III, IV, V	10/21	32.3/67.7
Pancreatic fistula**		
None, grade A/grade B, C	14/17	45.2/54.8
Liver infarction		
Yes/no	8/23	25.8/74.2
Postoperative hospital stay (days)	37 (16–182)	
30-day mortality		
Yes/no	0/31	0/100
90-day mortality		
Yes/no	2/29	6.5/93.5

^*^According to Clavien–Dindo (C–D) classification (ver. 2.0)

^**^According to International Study Group of Pancreatic Surgeons

**Fig. 3:**
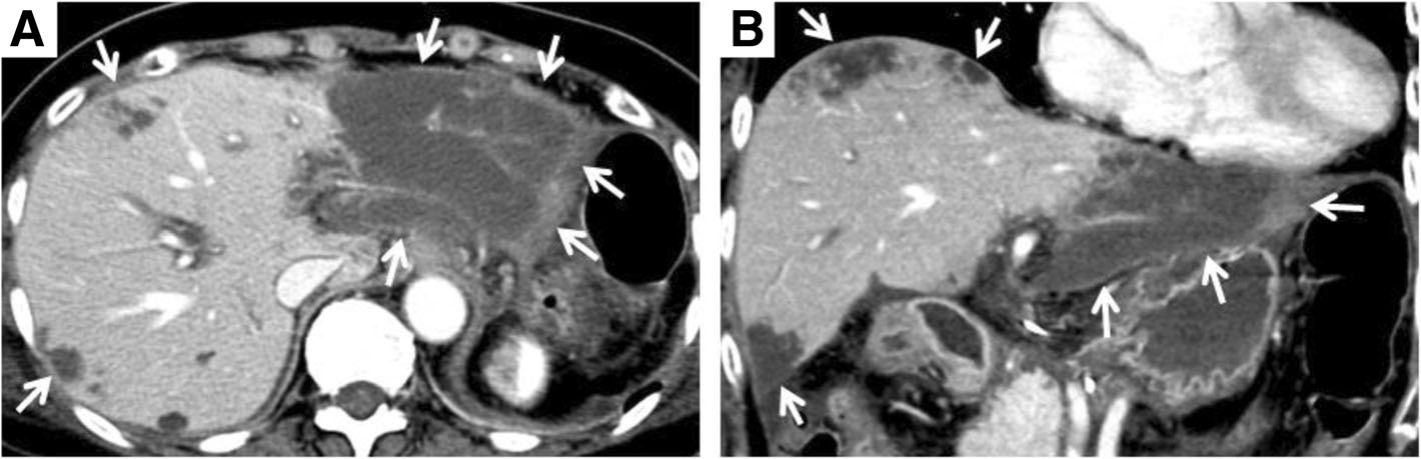
Axial (**a**) and coronal planes (**b**) of portal venous phase in MDCT of a representative case with postoperative liver infarction (white arrow). Infarction lesions detected in S5, S7, and S8 and whole lateral segments occupying 28.4% of the whole liver

Liver infarction developed only in 8 patients (25.8%). All lesions were detected by MDCT performed at postoperative day 7. The median of percent whole liver volume of the infarcted lesion was 9.3% (range, 2.1–28.4; Fig. 3). These lesions were not detected (i.e., recovered or disappeared) by MDCT 3–6 months after the surgery in all patients. Any liver-related complications other than liver infarction were not observed. To evaluate the effect of the preoperative coil embolization on the prevention of postoperative ischemic complications, we analyzed the correlation between preoperative coil embolization and the occurrence of liver infarction and other postoperative complications (Table 3). Seven patients (30.4%) developed postoperative liver infarction in the preoperative coil embolization group, whereas only one patient (12.5%) had postoperative liver infarction in the group without coil embolization. The patient without coil embolization developed a liver abscess in the lateral segment of the liver which required percutaneous transhepatic abscess drainage. Preoperative coil embolization was not associated with the occurrence of postoperative liver infarction (*p* = 0.642). Furthermore, duration from preoperative coil embolization to surgery was not associated with the occurrence of postoperative liver infarction (median, 15 days with liver infarction vs. 23 days without liver infarction, *p* = 0.203).

**Table 3:** Correlation of major complications and preoperative coil embolization

Factor	Preoperative coil embolization (+) (*n* = 23)	Preoperative coil embolization (−) (*n* = 8)	*p* value
Liver infarction			0.642
Yes	7	1	
No	16	7	
Pancreatic fistula*			1.000
None, grade A	10	4	
Grade B, C	13	4	
Chylous leakage			1.000
Yes	4	1	
No	19	7	
Morbidity**			1.000
C–D I, II	15	6	
C–D III, IV, V	8	2	
Postoperative hospital stay (days)	35.0 (16–182)	42.5 (24–68)	0.978

All results are shown as median (range) or number

*According to International Study Group of Pancreatic Surgeons

**According to Clavien–Dindo (C–D) classification (ver. 2.0)

To investigate risk factors for the postoperative liver infarction in all 31 patients who underwent DP-CAR, we analyzed the correlations between liver infarction and clinicopathological factors (Table 4). In terms of preoperative factors, the tumor size evaluated with MDCT was larger (median, 5.0 cm vs. 3.0 cm; *p* = 0.021) and tumor contact with the GDA/PHA on MDCT was more frequently observed (75.0% vs. 17.4%; *p* = 0.006) in the infarction group than in the no infarction group. Administration of NAC was not associated with the occurrence of liver infarction (*p* = 0.642). In terms of intraoperative factors, the infarction group had longer operative time (median, 434 min vs 298 min; *p* = 0.005) and performed concomitant PV resection and reconstruction more frequently (87.5% vs 39.1%; *p* = 0.037) than the no infarction group. In terms of postoperative factors, stenosis of the GDA/PHA on postoperative MDCT was observed in all patients in the infarction group and only 3 patients in the no infarction group (100.0% vs. 13.0%; *p* < 0.001). The GDA/PHA contact on MDCT was observed in 10 patients. Of these patients, 7 patients developed postoperative stenosis of the GDA/PHA. The incidence of the postoperative GDA/PHA stenosis was significantly high in patients with GDA/PHA contact compared to those without contact (*p* = 0.013). The length of postoperative hospital stay in the infarction group was longer than that in the no infarction group (median, 47.5 days vs. 34.0 days; *p* = 0.037).

**Table 4:** Correlation of clinicopathological factors and surgical outcomes of patients with or without postoperative liver infarction

Factor	Liver infarction (+) (*n* = 8)	Liver infarction (−) (*n* = 23)	*p* value
Age (years)	61.5 (42–78)	65.0 (39–80)	0.428
Gender, male/female	5/3	16/7	1.000
CEA (ng/mL)	4.0 (1.0–14.0)	3.3 (0.7–493.0)	0.519
CA19-9 (U/mL)	401.0 (3.3–16,860.0)	235.5 (0.1–3211.0)	0.075
Tumor size (MDCT) (cm)	5.0 (1.7–10.1)	3.0 (1.7–7.4)	0.021
GDA/PHA contact (MDCT), yes/no	6/2	4/19	0.006
Anomaly, yes/no	3/5	9/14	1.000
Neoadjuvant chemotherapy, yes/no	7/1	17/6	0.642
Preoperative coil embolization, yes/no	7/1	16/7	0.642
Duration since coiling to operation (days)	15.0 (4–87)	22.5 (4–104)	0.203
Operative time (min)	434 (313–584)	298 (175–540)	0.005
Blood loss (g)	2130 (365–5455)	1100 (305–10270)	0.448
Blood trans fusion, yes/no	5/3	12/11	0.698
Portal vein resection, yes/no	7/1	9/14	0.037
Gastrectomy, yes/no	3/5	2/21	0.093
Bile duct resection, yes/no	1/7	0/23	0.258
Postoperative portal vein stenosis, yes/no	5/3	6/17	0.095
GDA/PHA stenosis, yes/no	8/0	3/20	< 0.001
Tumor size (specimen) (cm)	5.6 (2.8–10.0)	4.0 (2.0–6.5)	0.022
Histologic type, well/mod./por./other	3/2/2/1	7/12/2/2	0.505
Lymph node metastasis, yes/no	7/1	17/6	0.642
Arterial invasion, yes/no	5/3	9/14	0.413
Portal vein invasion, yes/no	6/2	15/8	1.000
Perineural invasion, yes/no	7/1	22/1	0.456
Residual tumor status, R0/R1, 2	4/4	9/14	0.689
UICC stage (7th edition), IIA/IIB, III, IV	0/8	6/17	0.298
Morbidity*, C–D I, II/C–D III, IV, V	1/7	9/14	0.222
Pancreatic fistula**, None, grade A/grade b, C	3/5	11/12	0.698
Liver abscess, yes, no	1/7	0/23	0.258
Postoperative hospital stay (days)	47.5 (26–182)	34.0 (16–97)	0.037

All results are shown as median (range) or number

*UICC* Union for International Cancer Control

*According to Clavien–Dindo (C–D) classification (ver. 2.0)

**According to International Study Group of Pancreatic Surgeons

**Fig. 4:**
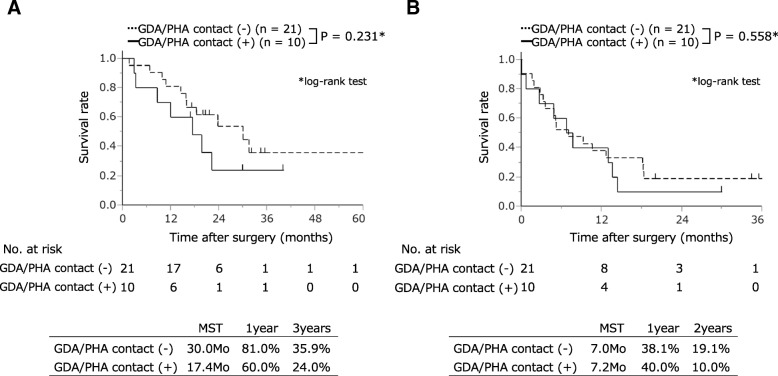
Long-term outcomes of patients grouped by the status of GDA/PHA surroundings, and presence or absence of tumor contact with these arteries. **a** Overall survival. **b** Disease-free survival

Preoperative tumor contact with the GDA/PHA was a risk factor of postoperative liver infarction. Thus, we examined the correlation between prognosis and tumor contact with the GDA/PHA on preoperative MDCT. The R0 resection rate in the cases with tumor contact was not significantly different from that in the cases without tumor contact (30.0% vs. 47.6%; *p* = 0.452). The MST and 1- and 3-year overall survival rates in the contact cases were 17.4 months and 60.0% (95% CI 29.7–84.2%) and 24.0% (95% CI 6.2–60.1%), respectively, whereas 30.0 months and 81.0% (95% CI 58.8–92.7%) and 35.9% (95% CI 15.6–62.9%) in cases without contact, respectively (Fig. 4a). The median time of disease-free survival and 1- and 2-year disease-free survival rates in the contact cases were 7.2 months and 40.0% (95% CI 15.8–70.3%) and 10.0% (95% CI 1.4–46.7%), respectively, whereas 7.0 months and 38.1% (95% CI 20.3–59.7%) and 19.1% (95% CI 7.3–41.2%) in cases without contact, respectively (Fig. 4b). No significant difference was found in the long-term result, overall survival, and disease-free survival between the two groups (*p* = 0.231, *p* = 0.558, respectively).

## Discussion

One of the major advantages of DP-CAR is that arterial reconstruction is not required, which can avoid the potentially life-threatening troubles after arterial reconstruction. However, DP-CAR has a theoretically significant risk of ischemic complications in the liver and stomach because of the combined resection of CHA and LGA. The ischemic complication in the liver rarely occurs after pancreatic resection, but it can cause a fatality when it happens. Hackert et al. reported the influence of liver ischemia on clinical outcome after pancreatic resection [18]. Postoperative blood flow insufficiency in the liver caused sepsis and multiple organ failure in 5 of 17 patients (29.4%) who had ischemic complications. The incidence of ischemic complication was reported as 2.2%. Similar findings are also reported in other studies [19]. The Johns Hopkins group reported the complication following PD in their 1000 consecutive cases. The postoperative mortality of PD was 1% (10/1000), and 20% of the mortality (2/10) resulted from liver necrosis and 10% (1/10) from liver abscess, most probably due to ischemic condition in the liver [20]. The Beaujon Hospital group reported 2.6% of postoperative mortality in a series of 545 patients who underwent PD. The incidence of ischemic complications was 1% (6/545), and 83.3% (5/6) patients died due to ischemic complications [19]. All these three reports suggested that liver perfusion failure is an important and underestimated risk factor for mortality following pancreatic resection.

In the current study, liver infarction occurred in 8 of 31 patients (25.8%) after DP-CAR. Among 8 patients, 7 patients had transient liver infarction and 1 patient, who had suffered from cholangitis preoperatively, developed liver abscess. Of 7 patients, 2 patients had prolonged fever and other patients did not have any liver-related symptoms. Liver infarction/abscess was significantly associated with long postoperative hospital stay, mostly due to prolonged fever and poor general condition. Additionally, the morbidity rate and R1 resection rate were relatively high in the current study. DP-CAR is potentially a highly invasive procedure with high morbidity, as reported in previous literature [21–26] (Table 5). Because our institution is one of the regional high-volume centers, many patients with highly advanced disease were referred to our institution. For such patients, it is sometimes necessary to perform extended resection, including concomitant PV resection, gastrectomy, bile duct resection, and/or retroperitoneal tissue excision. The high morbidity rate and R1 resection rate might be attributed to those extended resections due to highly advanced disease. Nevertheless, the postoperative mortality rate was comparable to that of reported series (Table 5), meaning that all complications could be managed safely, which may be, at least partly, due to the limited infarction area of the liver.

**Table 5:** Summary of recent studies and present series

Reference (year)	*n*	Preoperative coil embolization (%)	Morbidity, C–D III, IV (%)	Mortality (%)	Ischemic complication (%)	R0 (%)	Hospital stay (days)	Median survival (months)
Okada et al. (2018) [21]	50	92	34	8	Liver 62 Stomach 10	62	21	16
Klompmaker et al. (2018) [22]	68	HA 22.1	25	16.4	Liver 17.7 Stomach 4.4	54.6	16.5	17
Sugiura et al. (2017) [23]	16	n.a.	88	0	0	62	26	17.5
Sato et al. (2016) [24]	17	0	41	0	0	94	34	16.9
Nakamura et al. (2016) [25]	80	HA 100 LGA n.a.	36.3	5	Liver 10.1 Stomach 28.8	92.5	38	30.9
Oculin et al. (2016) [26]	30	n.a.	35	14	n.a.	80	10.7	35
Present series	31	74.2	61.3	6.5	Liver 25.8	41.9	37	23.7

*HA* hepatic artery, *LGA* left gastric artery, *n.a.* not available

Kondo et al [15]. first described preoperative coil embolization in DP-CAR to reduce the risk of postoperative ischemic complication. Subsequently, several authors have reported the feasibility and usefulness of this procedure [10, 16, 17]. Denecke et al. observed ischemic complications in the stomach only in patients who did not receive preoperative embolization of the CHA and LGA. They concluded that preoperative embolization of the celiac trunk, CHA, and LGA is helpful to avoid ischemic complications after DP-CAR [27]. However, coil embolization of the CHA is somewhat technically demanding and associated with potential risk of misplacement of coil in the PHA and/or GDA. Additionally, the cost of material for embolization, particularly the interlocking detachable coil, which is frequently used in the procedure, cannot be overlooked. Interestingly, in the present study, liver infarction and abscess formation were observed in patients who underwent preoperative coil embolization. However, no signs of liver ischemia were observed in 7 of 8 patients who did not undergo preoperative coil embolization. The most closely related risk factor of postoperative liver infarction was postoperative GDA/PHA stenosis (*p* < 0.001). All the 8 patients with liver infarction or abscess had GDA/PHA stenosis following DP-CAR. On the other hand, postoperative liver infarction was not developed in any of the 20 patients without stenosis. This result indicates that the maintenance of sufficient hepatic arterial flow through GDA is a key to avoid liver infarction irrespective to preoperative coil embolization. Of note, this result does not indicate that performing coil embolization caused a problem leading GDA/PHA stenosis, but the dissection along to the GDA/PHA did.

Larger tumor size and tumor contact with the GDA/PHA were preoperative risk factors of liver infarction and abscess. During operations in patients with these tumor characters, dissection along the GDA/PHA is necessary, which results in exposure of the long segment of GDA/PHA. This manipulation during operations could cause stenosis of the GDA/PHA, which was closely associated with liver infarction and abscess. These findings indicate that the complete preservation of GDA/PHA is important to maintain arterial blood flow in the liver. Even a slight decrease in blood flow in the GDA/PHA, which resulted from stenosis, may cause an ischemic condition in the liver. In addition, the presence of collateral arteries around the liver should be considered. There are various collateral pathways such as the bilateral inferior phrenic artery branched from the aorta nearby CeA and the accessory left hepatic artery branched from the LGA [28, 29]. Because these collateral arteries are frequently lost during dissection around the CeA and LGA, the presence of GDA/PHA is more important than the usual condition. Given these data, the most important technical aspect of reducing the risk of ischemic complications is the complete preservation of arterial flow of the GDA, regardless of preoperative coil embolization. Preoperative coil embolization of the CHA may not be mandatory to prevent ischemic liver complications.

Our inclusion criteria for DP-CAR are another concern. We indicated DP-CAR even for tumor contact with the GDA/PHA, which is one of the predicting factors of postoperative liver infarction in our series. Although the incidence of liver infarction was high in patients with GDA/PHA contact, all lesions were manageable and did not lead to fatal complications. The long-term survival was similar to patients without GDA/PHA contact, and the long-term outcome was comparable to that reported in previous literature (Table 5). Therefore, GDA/PHA contact is not a contraindication for DP-CAR, but meticulous attention is needed to avoid arterial injury during operation. Recently, preoperative chemoradiotherapy is frequently induced for patients with locally advanced PDAC [30, 31]. In our series, 77% of patients underwent preoperative chemotherapy. Accurate evaluation of the actual vascular invasion by MDCT is quite difficult due to therapy-induced regional changes [32]. Therefore, considerable caution is necessary to avoid overestimation of the degree of vascular invasion, particularly after chemoradiotherapy, which could lead to loss of a chance of surgical resection.

The current study has limitations. All data were collected retrospectively from the database of a single institute, and the sample size is small. Additionally, background data of patients are not matched, indicating that selection bias may occur. However, the current study analyzed clinical data of preoperative coil embolization and DP-CAR performed by a single team, meaning that the quality of each procedure was homogeneous throughout the study period. Because the quality of both coil embolization and DP-CAR could affect the postoperative outcome, our data are relevant to clinical practice in pancreatic surgery.

In conclusion, our data indicate that preoperative coil embolization of the CHA is not useful in DP-CAR as long as the GDA is completely preserved during surgery. A prospective study is essential to elucidate the necessity of preoperative coil embolization in the future.

## Data Availability

The datasets used during this study are available from the corresponding author on reasonable request.

## Abbreviations

DP-CAR: Distal pancreatectomy with en bloc celiac axis resection
PDAC: Pancreatic ductal adenocarcinoma
